# Characterization of Aryl Hydrocarbon Receptor Interacting Protein (AIP) Mutations in Familial Isolated Pituitary Adenoma Families

**DOI:** 10.1002/humu.21292

**Published:** 2010-08

**Authors:** Susana Igreja, Harvinder S Chahal, Peter King, Graeme B Bolger, Umasuthan Srirangalingam, Leonardo Guasti, J Paul Chapple, Giampaolo Trivellin, Maria Gueorguiev, Katie Guegan, Karen Stals, Bernard Khoo, Ajith V Kumar, Sian Ellard, Ashley B Grossman, Márta Korbonits, the International FIPA Consortium

**Affiliations:** 1 Department of Endocrinology, Barts and the London School of Medicine, Queen Mary University of LondonLondon, United Kingdom; 2 Comprehensive Cancer Center, University of AlabamaBirmingham, Alabama; 3 Department of Molecular Genetics, Royal Devon and Exeter Foundation TrustExeter, United Kingdom; 4 Department of Endocrinology, UCL Medical School, Royal Free CampusLondon, United Kingdom; 5 North East Thames Regional Genetics Service, Great Ormond Street HospitalLondon, WC1N 3JH, United Kingdom

**Keywords:** pituitary adenoma, FIPA, acromegaly, AIP, tumor suppressor

## Abstract

Familial isolated pituitary adenoma (FIPA) is an autosomal dominant condition with variable genetic background and incomplete penetrance. Germline mutations of the aryl hydrocarbon receptor interacting protein (*AIP*) gene have been reported in 15–40% of FIPA patients. Limited data are available on the functional consequences of the mutations or regarding the regulation of the *AIP* gene. We describe a large cohort of FIPA families and characterize missense and silent mutations using minigene constructs, luciferase and β-galactosidase assays, as well as in silico predictions. Patients with *AIP* mutations had a lower mean age at diagnosis (23.6±11.2 years) than AIP mutation-negative patients (40.4±14.5 years). A promoter mutation showed reduced in vitro activity corresponding to lower mRNA expression in patient samples. Stimulation of the protein kinase A-pathway positively regulates the *AIP* promoter. Silent mutations led to abnormal splicing resulting in truncated protein or reduced *AIP* expression. A two-hybrid assay of protein–protein interaction of all missense variants showed variable disruption of AIP-phosphodiesterase-4A5 binding. In summary, exonic, promoter, splice-site, and large deletion mutations in AIP are implicated in 31% of families in our FIPA cohort. Functional characterization of *AIP* changes is important to identify the functional impact of gene sequence variants. Hum Mutat 31:1–11, 2010. © 2010 Wiley-Liss, Inc.

## Introduction

The majority of pituitary adenomas are sporadic, but occasionally they may also occur in a familial setting [Melmed, 2006]. Familial isolated pituitary adenoma (FIPA; MIM♯ 102200) is an increasingly recognized autosomal dominant disease with low or variable penetrance [Beckers and Daly, 2007; Chahal et al., 2010]. Heterozygous germline mutations in the aryl hydrocarbon receptor-interacting protein (*AIP*; MIM♯ 605555) have been identified in 15–40% of FIPA families, whereas in the majority of the families the disease-causing gene or genes are not known. *AIP* mutation-positive families most commonly present with somato-troph, or with both somatotroph and lactotroph adenomas, but rarely other types of pituitary tumors can be observed [Daly et al., 2007; Iwata et al., 2007; Leontiou et al., 2008; Toledo et al., 2007; Vierimaa et al., 2006]. Young onset, seemingly sporadic somatotroph adenoma patients can also harbor germline *AIP* mutations [Georgitsi et al., 2008a]. Patients have loss of heterozygosity (LOH) in the tumor tissue at the locus of the *AIP* gene at the 11q13 area [Gadelha et al., 2000; Soares et al., 2005; Vierimaa et al., 2006; Yamada et al., 1997]. However, LOH at 11q13 has been observed frequently in sporadic pituitary adenomas [Farrell and Clayton, 2000], and there are a number of FIPA families with 11q13 LOH, but no detectable *AIP* mutations [Leontiou et al., 2008; Soares et al., 2005]. Interestingly, the 11q13 locus also contains the Multiple Endocrine Neoplasia type 1 gene (*MEN1*; MIM♯ 131100), 2.4 megabases upstream from the *AIP* gene, which can cause familial pituitary adenomas; however, the phenotype of MEN1 and FIPA is different.

The *AIP* gene consists of six exons encoding a 330 amino acid protein with three typical tetratricopeptide repeat (TPR) domains and a final extended α-helix (α-7). *AIP* appears to function as a tumor suppressor gene: we have previously reported data showing that mutant AIP proteins lose the ability of wild-type (WT) AIP to decrease cell proliferation and are unable to bind protein partners [Leontiou et al., 2008]. Most of the described *AIP* mutations identified via standard sequencing techniques change the amino acid sequence due to nonsense, deletion, or insertion mutations, and result in a loss of the C-terminal end of the protein. Large gene deletions also severely disrupt the protein structure. Exon/intron junction mutations may affect splicing or RNA stability and promoter mutations affect RNA expression, but these have not been previously characterized.

We have systematically examined our large cohort of patients with FIPA specifically looking for large deletions, promoter, and splice-site mutations in addition to exonic mutations; we describe 11 new *AIP* mutation-positive families with six novel mutations. We present functional data on an *AIP* promoter mutation, a splice-mutation and a synonymous change, which leads to reduced *AIP* mRNA expression, as well as quantitative data on the effects of all the published missense mutations on the interaction of AIP with one of its binding proteins, phosphodiesterase-4A5 (PDE4A5). These data allow us to characterize the clinical features of FIPA and to provide the most accurate data for the functional impact of *AIP* variants, which has an important role in genetic counseling of patients and their families and will help inform the need for predictive testing and biochemical and imaging screening.

## Patients and Methods

### FIPA Patients

We studied 38 novel families with FIPA (Supp. Table S1) who were identified as having at least two family members with pituitary adenoma and no features of MEN1 or Carney complex (MIM♯ 160980). All patients provided written informed consent, and institutional review board approval was obtained. For the clinical analysis we combined these novel families with our previously described cohort of 26 families (Supp. Table S2) [Leontiou et al., 2008]. The clinical features of some of these families have previously been described, as detailed in Supp. Table S1 and S2 [Gadelha et al., 1999, 2000; Georgitsi et al., 2007, 2008b; Leontiou et al., 2008; Matsuno et al., 1994; McCarthy et al., 1990; Pestell et al., 1989; Soares et al., 2005]. Direct sequencing of AIP (NM_003977.2) included the entire coding sequence, conserved splice sites (from the conserved A of the upstream branch site to + 10 downstream of each exon) and 1,200 base pairs of the promoter region. Nucleotide numbering throughout the manuscript reflects cDNA numbering with 11 corresponding to the A of the ATG translation initiation codon in the reference sequence, according to the guidelines of the Human Genome Variation Society (http://www.hgvs.org/mutnomen). The initiation codon is codon 1. *AIP* sequencing data were compared with Caucasian (n 5 96) and Japanese (*n* =78) subjects from the general population, as previously described [Leontiou et al., 2008]. Multiplex ligation-dependent probe amplification (MLPA, P244-kit MRC-Holland, Amsterdam, The Netherlands) dosage analysis was carried out to look for partial or whole gene deletions in all the families that tested negative by direct sequencing for germline *AIP* mutations and for whom a suitable quality of DNA sample was available.

### RNA Extraction, RT-PCR, Cell Culture

RNA from whole blood was extracted using PaxGene tube and extraction kit (Qiagen, Crawley, UK). RNA from rat pituitary cell line GH3 was extracted using the RNeasy Mini Kit (Qiagen). Both protocols include a DNase step. RT-PCR on 1μg RNA was performed as described previously [Leontiou et al., 2008]. Realtime PCR was performed with the TaqMan system using ready-made or custom-made (surrounding the c.807C > T mutation) AIP probe-primer kits (ABI, Warrington, UK). Reactions were performed in triplicate using β-actin or GAPDH as a housekeeping gene. Data were analyzed using the standard curve method. Rat somatomammotroph GH3 cell line was cultured and transfected as previously described [Leontiou et al., 2008].

### Promoter Mutation

**Figure 1:**
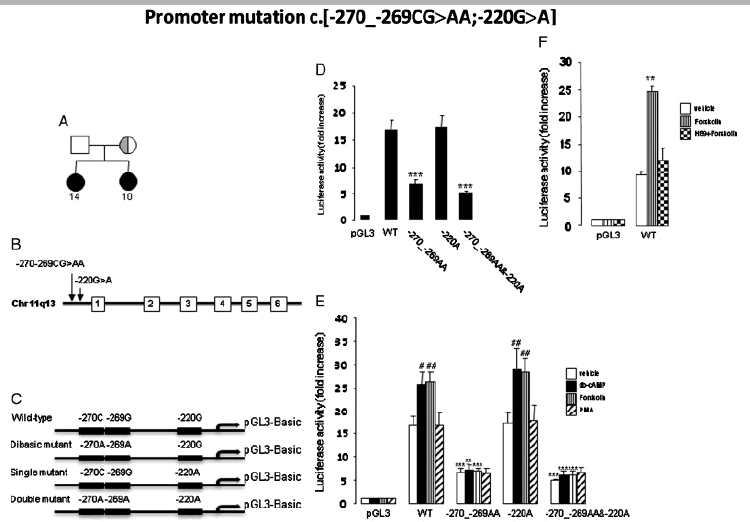
AIP promoter mutations. **A:** Family tree (Family VI, Supp. Table S2), filled circles represent patients with gigantism with age of onset shown, half-filled symbols represents carrier subjects. **B:** Location of the two *AIP* promoter sequence changes. Numbers in boxes represent exon numbers. Nucleotide numbering reflects cDNA numbering with +1 corresponding to the A of the ATG translation initiation codon in the reference sequence. **C:** Schematic representation of the four AIP promoter constructs. **D:** The wild-type (WT) and the c.−220A single mutation showed similar promoter activity measured by luciferase assay. The −270_−269AA dibasic mutant and the double −270_−269AA and −220A mutant constructs showed decreased promoter activity (***P<0.001 vs. WT). **E:** AIP promoter activity after treatment with db-cAMP, forskolin, and PMA. The WT and the −220A single mutant constructs showed increased promoter activity after treatment with db-cAMP and forskolin compared to vehicle treatment (♯*P* < 0.05, ♯♯*P* < 0.01). Treatments did not affect the promoter activity of the dibasic −270_−269AA and double −270 −269AA and −220A mutant constructs. Following db-cAMP and forskolin there was a significantly lower promoter activity in the −270_−269AA and −270 −269AA and −220A mutant constructs compared to WT (***P < 0.001, **P < 0.01 vs. WT). **F:** The PKA inhibitor H89 inhibits the stimulating effect of forskolin on luciferase activity of the WT-promoter.

The c.[−270_−269CG > AA;−220G > A] changes in the promoter area were identified in a Japanese somatotroph adenoma family (Family VI Supp. Table S2). These changes were not detected in Japanese (*n* =78) and Caucasian (*n* =96) individuals from the general population or in any of the studied family members (affected or unaffected *AIP* mutation carriers or their noncarrier family members or AIP mutation negative patients, *n* =150) of our familial cohort, or in any of our sporadic pituitary adenoma cases studied (*n* = 98), altogether 844 chromosomes. The transcription factor binding-site searching programs TESS (http://www.cbil.upenn.edu/cgi-bin/tess/tess) and Alibaba (http://www.gene-regulation.com/pub/programs/alibaba2/index.html) were used to identify possible binding site disruption caused by the c.[−270_−269CG > AA;−220G > A] sequence changes in the *AIP* promoter area (Fig. 1A and B). A 1.2-kb (upstream from the start codon) PCR fragment was amplified from gDNA (forward 5′TACAACCTCCATCTCCTGGG3′, reverse 5′GAGTCCGGAAGTTG-CCGAAA3′). The WT fragment was cloned into the *SacI* and *Spe*I sites of the promoterless firefly luciferase vector pGL3-basic (Promega, Southampton, UK). Three mutated constructs were prepared from the WT template using the QuikChange site-directed mutagenesis kit (Stratagene, LaJolla, CA): a dibasic −270_−269AA mutant, a single −220A mutant and a construct containing both −270_−269AA and −220A mutations (Fig. 1C). To study basal and stimulated activity, GH3 cells were transfected with the four different constructs and 48 hr later were treated with db-cAMP (2 mM), forskolin (10 μM), phorbol-12-myristate 13-acetate (PMA, 20 nM) and H89 (30 μM) for 5hr. A plasmid encoding renilla luciferase, pRL-CMV, was included to control for transfection efficiency and promoter activity was analyzed using the dual luciferase assay (Promega). Peripheral blood-derived cDNA samples were studied from the two affected patients and three ethnically matched controls.

### Splicing Mutation c.249G > T

**Figure 2:**
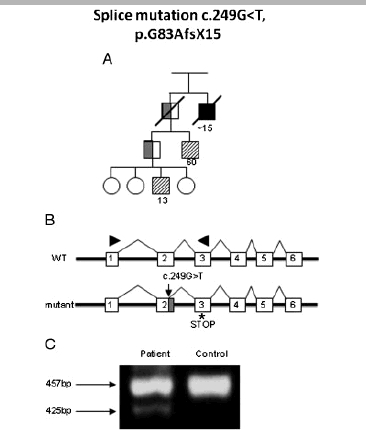
Alternatively spliced AIP transcript in the presence of the 249G > T mutation. **A:** Family tree (Family 28, Supp. Table S1) showing patients with gigantism (filled square), prolactinoma (striped squares), and carrier subjects (half-filled symbols) with age of onset shown. **B:** Schematic representation of splicing in the WTand mutant gene showing the location of the mutation (arrow) and the 32 bp lost from the end of exon 2 (shaded area) followed by a novel stop-codon after 15 novel codons (marked with *). Numbers in boxes represent exon numbers. Primers used are shown by arrowheads. **C:** RT-PCR using a patient and control cDNA. Patient cDNA shows an extra band (425 bp) that corresponds to the alternatively spliced AIP transcript with the upper band showing the WT transcript (457 bp). The identity of these PCR products were confirmed by sequencing.

The ALAMUT program (http://www.interactive-biosoftware.-com/alamut/doc/1.5/splicing.html) was used to predict whether the novel synonymous sequence variant c.249G > T, *p.* = (affecting the third nucleotide of codon 83 originally coding glycine) changes the splicing characteristics of the *AIP* gene (Family 28, Fig. 2A). The primers (forward 5′GCGGATATCATCGCAAGACT3′, reverse 5′CCTCATCTTCCACATGGAGA3′) were designed on exons 1 and 3 to detect aberrant splicing events.

### Synonymous Mutation c.807C4 > T

**Figure 3:**
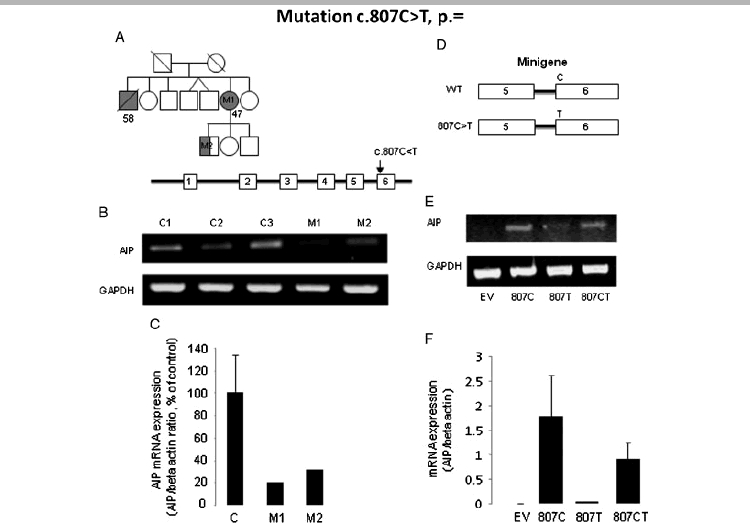
Decreased *AIP* mRNA expression in the presence of the 807C > T. **A:** Family tree showing two patients with acromegaly and an asymptomatic carrier with age of onset of disease (Family XVI, Supp. Table S2). The location of the mutation is shown by an arrow; numbers in boxes represent exon numbers. **B:** Conventional RT-PCR with AIP primers on blood-derived cDNA obtained from three control individuals (C1–3) and two subjects (M1 and 2) carrying the 807C > T AIP mutation. Patients carrying the 807C > T showed decreased *AIP* expression. **C:** Real-time PCR using an AIP primer and probe set to compare the *AIP* levels between control individuals and patients; C (control). **D:** Wild-type (WT) and mutant minigene constructs. **E:** Conventional RT-PCR using vector-specific primers for the minigene constructs showing decreased mutated minigene expression (807T) and intermediate level of expression for the coexpression of WTand mutant minigenes (807CT). **F:** Real-time PCR showing an increased AIP expression in the WT minigene construct compared to mutant and coexpression of WTand mutant minigenes. EV, empty vector.

The ALAMUT, the ESEfinder v.3.0 (http://rulai.cshl.edu/cgi-bin/tools/ESE3/esefinder.cgi?process=home) and the RESCUE-ESE (http://genes.mit.edu/burgelab/rescue-ese/) programs were used to predict whether the synonymous c.807C > T, *p*. = (affecting the third member of codon 269 originally coding phenylalanine) sequence variant of exon 6 changes the splicing characteristics of the AIP gene (Fig. 3A) [Leontiou et al., 2008]. Conventional and real-time RT-PCRs were performed on patient blood-derived cDNA using primers spanning the exon 5–6 junction to compare AIP expression between patients carrying the mutation and control individuals. To further characterize the splicing events around this sequence abnormality we performed a minigene splicing study. To construct the minigene, gDNA from a patient harboring the c.807C > T mutation was PCR amplified with primers covering part of the exon 5, intron–exon region and part of exon 6 including the mutation (forward 5′AAGCTGGTGGTCGAGGAGTA3′, reverse 5′CAAAGTGCTGGAGCTGGAC3′). WT and mutant minigene constructs were prepared and fragments were cloned into the *EcoR*I site of pcDNA3.1+ (Invitrogen, Paisley, UK). GH3 cells were transiently transfected for 24–48 hr with WT and mutant minigenes separately or together in equal amounts. Transfection efficiency was followed by cotransfection with an expression plasmid coding for green fluorescent protein (GFP, pEGFP-N1) at a pcDNA3.1+with insert:pEGFP-N1ratio of 1:10. Expression of the minigene transcripts was detected using vector-specific primers.

### PDE4A5 Interaction

Interactions between AIP and PDE4A5 were studied to clarify the role of missense sequence changes in protein-protein binding. *PDE4A5* was cloned into the *Not*I-site of pLEXAN to generate a LexA DNA-binding-domain fusion [Bolger et al., 2003; Leontiou et al., 2008]. WT *AIP* and nine missense *AIP* sequence variants (c.47G > A, p.R16H [Buchbinder et al., 2008; Cazabat et al., 2007; Daly et al., 2007; Georgitsi et al., 2007; Yaneva et al., 2008]; c.145G > A, p.V49M [Iwata et al., 2007]; c.308A > G, p.K103R [Beckers et al., 2008], c.713G > A, p.C238Y [Leontiou et al., 2008], c.721A > G, p.K241E [Daly et al., 2007], c.769A > G, p.I257V [Montanana et al., 2009], c.811C > T, p.R271W [Daly et al., 2007; Igreja et al., 2010; Jennings et al., 2009], c.896C > T, p.A299V [Georgitsi et al., 2007], the hotspot mutation c.911G > A, p.R304Q, and a novel stop mutation (c.490C > T, p.Q164X) were cloned into the NotI-site of pGADN to generate GAL4 activation-domain fusions. Quantitative β-galactosidase assays were performed in the *Saccharomyces cerevisiae* strain L40 by the method of Guarente [1983] using O-nitrophenyl-β-D-galactopyranoside as a substrate. Each mutation was tested in at least two different yeast clones.

### In Silico Analysis

Location of *AIP* missense mutations were compared to the available consensus sequences of TPR proteins. A hypothetical model of AIP was constructed using the Phyre (Protein Homology/analogY Recognition Engine) program [Kelley and Sternberg, 2009], based on the crystal structure of FKBP51 (MIM♯ 602623), the closest related protein with available crystal structure (c1kt0A). The alignment is showing 22% identical amino acids and 45% similar amino acids in the N-terminal region (amino acids 3–90 of AIP) and the TPR domain area (amino acids 165–328 of AIP) (Supp. Fig. S1). Alignment was performed with CLUSTAL 2.0.12 (http://www.clustal.org/) and BL2SEQ (http://www.ncbi.nlm.nih.gov/blast/bl2seq/wblast2.cgi) sequence alignment tools.

### Statistics

Statistical analysis was performed with StatsDirect software (Addison-Wesley-Longman, Cambridge, UK). Distribution of data was analyzed by the Shapiro-Wilk test. Comparisons were calculated with the Student *t*-test and the Kruskal-Wallis test followed by the Conover-Inman test, as appropriate. Data are shown as mean ± standard error, unless otherwise stated. Significance was taken at *P* < 0.05.

## Results

### Genetic Studies

We report here 38 novel families with FIPA, including 11 families with an *AIP* mutation (Supp. Table S1). Six of these 11 families harbor a novel mutation including two complete gene deletions.

The novel mutations revealed via sequencing include a nonsense mutation (c.490C > T, p.Q164X), a single base-pair duplication (c.662dupC, p.E222X), a splice-site mutation (c.249G > T, p.G83AfsX15, see data below) and a complex deletion-insertion mutation (c.74_81delins7, p.L25PfsX130), all resulting in a frameshift and/or premature stop-codon. Three families have been found to have the previously well-described c.910C > T, p.R304X hotspot [Daly et al., 2007; Jaffrain-Rea et al., 2009; Leontiou et al., 2008; Vierimaa et al., 2006] mutation and one family the c.911G > A, p.R304Q hotspot [Daly et al., 2007; Leontiou et al., 2008] mutation. We also identified a previously described variant (c.896C > T, p.A299V) in one of our families that also harbors the p.R304X mutation.

We screened all affected patients in our cohort (Supp. Tables S1 and S2) for large deletions using MLPA, where no AIP mutations were identified by sequencing and suitable DNA was available. Three large deletions were identified among the 36 families (8.3%, Family 9, Family IV, and Family XXI; Supp. Tables S1 and S2), all three originally reported to have WT AIP sequences by conventional sequencing [Georgitsi et al., 2007; Leontiou et al., 2008]. Two of these resulted in the loss of the full length *AIP* gene and one was the previously described exon 2 deletion [Georgitsi et al., 2008b].

For clinical characteristics we analyzed the data from the currently reported 38 families together with the 26 families we have reported earlier [Leontiou et al., 2008]. In our cohort of 64 FIPA families with 160 affected subjects (Supp. Tables S1 and S2), the mean age (±SD) at diagnosis of pituitary adenoma was 33.7 ± 15.5 years (23.6 ± 11.2 years in the AIP mutant families and 40.4 ± 14.5 years in the AIP negative families; *P* < 0.00001). If we recalculate the age of onset only for the successfully performed MLPA negative families this was 38.4 ± 13.8 years (not significantly different from the full cohort of AIP negative families). Families with FIPA show variable penetrance. We are fully aware that penetrance calculations are subject to bias due to symptomatic patient referral and due to incomplete genealogical data as well as variable ages of the subjects studied, but these calculations can still provide some useful information. Penetrance was calculated in our families with taking into account affected and obligate carrier subjects as well as half of the subjects with 50% risk to inherit the mutation [Ogino et al., 2007]. We have a mean ± SD penetrance of 42 ± 21% for AIP-positive families with a minimum of 8% and a maximum of 83%. The penetrance in the *AIP* negative families was not significantly lower at 38 ± 16%, minimum 19% and maximum 67%. However, we observed a difference in the reported number of affected patients between *AIP* mutation-positive and -negative families, suggesting a difference in penetrance: the mean number of affected subjects in *AIP* mutation-positive families was 3.2 ± 1.8, whereas in *AIP* mutation-negative families it was 2.2 ± 0.4 (*P* < 0.001). Interestingly, 16 of our 20 families with an *AIP* mutation had at least one member with gigantism and/or disease onset < 18 years, whereas only 3 of our 44 AIP-negative FIPA families had a member with gigantism and/or disease onset < 18 years. In the 20 *AIP-*positive families 7 of the 63 affected subjects (11%) were diagnosed above the age of 35 years, three out of these (5%) were above 50 years. In the 44 *AIP*-negative families 56 of 96 patients (58%) were diagnosed above the age of 35 years, 24 of these (25%) above the age of 50 years. Five of 63 AIP mutation patients (8%) presented with pituitary apoplexy; three of them were in childhood. We studied whether the type of *AIP* mutation (presence or absence of C-terminal end of the protein) would influence penetrance. We have 16 families with mutations resulting in no protein or truncated proteins having 3.43 ± 1.9 patients per family and four families with full-length proteins having 2.5 ± 0.58 patients per family (no significant difference), suggesting the lack of genotype–phenotype correlation in these 20 *AIP*-positive families in terms of penetrance. Out of the 64 families, 59 had at least one member with a prolactinoma or acromegaly, whereas five families had pure nonfunctioning adenomas. Out of the 63 AIP mutation patients only 6 had “pure” prolactinoma, and all of these were macroadenomas. The AIP mutation-positive patients who clinically presented with a nonfunctioning adenoma all had positive GH and/or PRL staining in the adenoma tissue, suggesting that all subjects with *AIP* mutations have GH or PRL-synthesizing adenomas in our cohort.

### *AIP* Promoter Mutation Causes Decreased Promoter Activity

Two mutations (c.[−270_−269CG>AA; −220G > A] in cis) had been identified in a Japanese FIPA family with two sisters suffering from gigantism (Family VII; Supp. Table S2, Fig. 1A and B) [Leontiou et al., 2008]. These changes are located 160 and 111 bp upstream of the 5′UTR. The transcription factor binding-site searching programs suggested that the −270_−269CG > AA promoter mutation causes disruption of several transcription factor binding sites. To investigate whether the observed sequence changes affected the transcriptional activity of the *AIP* promoter, we cloned a 1.2-kb upstream fragment of the *AIP* gene into the pGL3-basic luciferase reporter vector (WT construct). To identify which of the mutations were functional, promoter constructs were prepared with a dibasic change (−270_−269AA), a single base change (−220A), and a construct with both changes (−270_−269AA and −220A) (Fig. 1C). These were transiently transfected into GH3 cells and the activity of the promoter was measured using a luciferase assay.

Transfection of the WT promoter and the single −220A promoter construct resulted in an 18-fold increase of promoter activity compared to empty vector pGL3-basic (Fig. 1D). Compared to the WT promoter construct the dibasic −270_−269AA promoter construct and the double −270_−269AA and −220A promoter construct had significantly decreased promoter activities (*P* < 0.001). These results suggest that the dibasic –270_−269AA promoter mutation, but not the single −220A variant, reduces promoter activity, which is likely to have a functional impact in the patients carrying this dibasic mutation, and suggests that this area is important for the regulation of the *AIP* promoter.

Additional support for the reduced function of this *AIP* promoter was generated with real-time PCR study of the two affected subjects' leukocyte cDNA (heterozygote for the promoter mutation) compared to three healthy controls from the same ethnic background. This showed reduced AIP expression (AIP/GAPDH mRNA ratio, mean ± SEM, 0.86 ± 0.2 vs. 0.58 ± 0.1, *P* = 0.05), supporting the reduced activity of the *AIP* promoter in these cells.

### *AIP* Promoter is Regulated via the cAMP-PKA Signaling Pathway

The cyclic AMP pathway is important for somatotroph pathogenesis as its upregulation via somatic *gsp* mutations (MIM♯ 139320) or via *PRKAR1A* (MIM♯ 188830) mutations lead to somatotropinomas [Horvath and Stratakis, 2008]. cAMP stimulates AIP binding partner AhR [Oesch-Bartlomowicz et al., 2005]. In addition, the extracellular signal-regulated kinase (ERK) pathway has recently been shown to be deregulated in pituitary adenomas [Dworakowska et al., 2009]. No previous data are available about the regulation of the *AIP* promoter. We therefore treated GH3 cells transfected with the 1.2-kb AIP promoter constructs with db-cAMP (a cAMP analog which activates PKA), forskolin (an adenylate cyclase activator that activates PKA), and PMA (a protein kinase C activator leading to activation of the ERK/MAPK pathway) and measured luciferase activity. Treatment with db-cAMP and forskolin increased WT and −220A promoter activity compared to vehicle (Fig. 1E). The effect of forskolin on the WT promoter was inhibited by the PKA inhibitor H89 (Fig. 1F). However, db-cAMP and forskolin treatment did not have any effect on the dibasic −270_−269AA mutated promoter or the double −270_−269AA and −220A mutated promoter. Treatment with PMA did not alter *AIP* promoter activity in any of the four promoter constructs. These results implicate the involvement of the cAMP–PKA signaling pathway in regulating *AIP* expression, and suggest that the –270_–269 region is required for cAMP-induced AIP promoter activity.

### Splice Mutation c.249G > T Causes Truncated AIP

A novel mutation (c.249G > T) was identified in a family (Family 28; Supp. Table S1) with two childhood-onset pituitary adenomas (a somatotrophinoma and a prolactinoma) and an adult-onset prolactinoma (Fig. 2A). The mutation at the end of exon 2 did not change the amino acid sequence, but in silico analysis using ALAMUT predicted that this mutation created a novel 5′ splice-site 32 bases upstream from the normal splice site resulting in a frameshift and a stop-codon after 35 novel amino acids (Fig. 2B). To determine whether this mutation indeed causes alternative splicing, we extracted RNA from blood of one of the patients. RT-PCR with primers upstream and downstream of the mutation was performed and compared to cDNA from a control individual. RT-PCR of cDNA from the patient heterozygous for the mutation generated the expected WT product plus an additional band that corresponded to the size of the predicted alternative product (425 bp; Fig. 2C). Sequencing of this smaller product confirmed the identity of the alternatively spliced transcript. These results confirmed the in silico prediction that the nucleotide change c.249G > T causes alternative splicing leading to a frameshift and a truncated AIP protein (p.G83AfsX15).

### The Synonymous c.807C > T Mutation Results in Reduced mRNA Expression

The mutation c.807C > T, *p*. = at the beginning of exon 6 had been identified in a family with two somatotrophinomas (Family XVI; Supp. Table S2) [Leontiou et al., 2008]. In silico analysis (ESEfinder and RESCUE-ESE) suggested that the c.807C > T mutation might result in the loss of a binding site for splice enhancers SRp40 and SRp55 (splicing regulatory proteins that recognize exonic splicing enhancer sequences) in the final exon of AIP (Fig. 3A). We explored whether this mutation affected the splicing of the last *AIP* exon. cDNA obtained from affected or carrier individuals was amplified with different primer sets covering the various sections of the exon 5–3′UTR region and compared to the WT gene. PCR amplification with the different primer sets did not show any novel alternatively spliced transcripts, but instead, patients with the mutation showed decreased *AIP* mRNA expression compared to control individuals using both conventional (Fig. 3B) and real-time PCR (Fig. 3C). Thus, these data suggests that the c.807C > T mutation may have a functional impact by reducing *AIP* transcript levels.

To further investigate whether the c.807C > T mutation leads to decreased mRNA expression, we performed in vitro studies using a minigene construct. The minigene was constructed with an amplified DNA fragment from a patient carrying the mutation. AWT (807C) and a mutated (807T) minigene including the 3′ end of exon 5, the intron between exon 5 and 6, and the 5′-end of exon 6 were cloned into the pcDNA3.1(+) expression vector (Fig. 3D). The WT minigene and the mutated minigene were then transiently transfected into GH3 cells for 48 hr. To mimic the patient's heterozygous mutation, cells were cotransfected with equal amounts of the WT and mutated minigenes. Using conventional (Fig. 3E) and real-time PCR (Fig. 3F), we detected decreased transcriptional products of the minigene in the cells transfected with the mutated minigene (807T) and an intermediate amount with the cotransfection of WT and mutated minigenes compared to cells with the WT minigene construct. These data corroborate the in vivo data showing that the c.807C > T variant is associated with reduced levels of *AIP* mRNA, supporting the functional impact of the mutation.

### Does the c.896C > T, p.A299V Variant have a Functional Impact?

**Figure 4:**
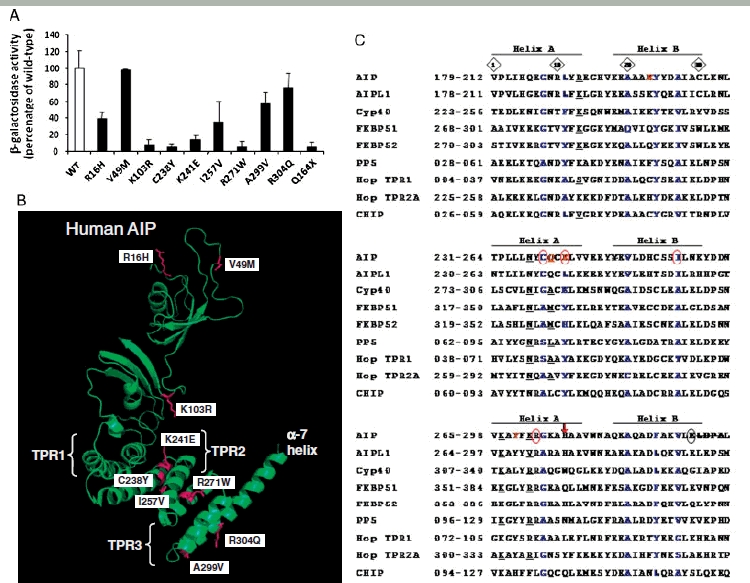
Single amino acid substitutions in AIP. **A:** A yeast two-hybrid quantitative β-galactosidase assay was used to assess the interaction of AIP with PDE4A5. It shows more than fivefold difference (activity 0–20% of wild-type [WT]) from WT AIP for missense mutations K103R, C238Y, K241E, and R271Was well as for the positive control truncation mutation Q164X. The R16H, V49M, I257V, A299V, and the R304Q variants show no difference or activity 33–100% of wild type (less than threefold difference) from WT AIP (mean ± SD). β-Galactosidase activity was measured as described by Guarente (1983). Each mutation was tested in at least two different yeast clones, with identical results (*n* = 3 for each clone). **B:** Hypothetical structure of AIP based on the structure of FKBP51 showing the three tetratricopeptide (TPR) domains with three pair of antiparallel α-helices and the final extended α-helix, α-7 (courtesy of Prof. David Barford, London, UK). Sequence comparison of human AIP and FKBP51 is shown in Supp. Figure S1. Amino acids with reported missense variants are highlighted. **C:** The three TPR domains, each consisting of an A and B helix, are shown of several Hsp90 binding proteins including AIP (table modified from Hidalgo-de-Quintana et al. [2008]). Numbers in diamond shapes show TPR motif position numbers. Amino acids marked with blue bold letters are important for the packaging and stability of the α-helices. Amino acids at position 8 and 20 were shown to be important in helix A and B packaging, while position 27 helps packaging of helix B with helix A of the same TPR domain and helix A of the following TPR domain. Underlined amino acids are predicted to be involved in the peptide binding pocket of FKBP51. Full-length variants of AIP affecting the TPR domains are shown on the figure (see review [Tahir et al., 2010]). Amino acids circled with red in the AIP sequence show missense variants described (C238Y, K241E, I257V, R271W). Amino acids marked with orange bold italics have been shown to be replaced by stop codon in FIPA patients (K201X, Q239X, K241X, Y268X). The amino acid marked by a red arrow is followed by an in-frame insertion (p.F269_H275dup) in a FIPA family. In-frame deletion variants p.Y238del is shown by strikethrough and complex missense and in-frame deletion mutation p.[E293G; L294_A297del] is shown by black circle and strikethrough.

In Family 10, two *AIP* changes were identified in two asymptomatic male carriers (70 and 65 years): the c.910C > T, p.R304X (a known pathogenic mutation) [Leontiou et al., 2008] and the c.896C > T, p.A299V (previously reported in a patient with sporadic acromegaly [Georgitsi et al., 2007], but in none of the general population subjects studied by us or others) variants. Other family members showed only the c.910C > T or the c.896C > T change. We cloned this region of the *AIP* gene from the gDNA of a subject carrying both mutations and sequenced several colonies. Some of the colonies carried the c.910C > T change while others the c.896C > T change. These data confirm that the two sequence changes reside on different chromosomes (in trans), and therefore the asymptomatic subjects are compound heterozygotes for these two AIP changes. Interestingly, one of the female carriers of the p.A299V variant, but not the p.R304X mutation, was diagnosed with a microprolactinoma at the age 30 years. Out of our seven “pure” prolactinoma patients found in our *AIP* mutation-positive families she is the only one with a microprolactinoma, and most of the others needed surgery and radiotherapy. We consider that her case might be a phenocopy, similar to the phenocopies reported in the Finnish Q14X mutation family [Vierimaa et al., 2006], however, as she carries the p.A299V *AIP* variant this is uncertain. We performed a functional assay to study the interaction of AIP and PDE > A5. The p.A299V variant of AIP did not show a profoundly reduced binding to PDE > A5 compared to WT controls (Fig. 4A). In summary, the clinical data on the compound heterozygote subjects support that the c.896C > T, p.A299V change might be a rare polymorphism and the in vitro data are compatible with this.

### Analysis of Missense *AIP* Mutations

We decided to study the protein–protein interaction properties of all missense mutations reported in the literature. We have previously shown with a filter β-galactosidase assay that the R81X, R217X, and R304X nonsense mutations disrupt the binding between AIP and PDE > A5. Here we used a quantitative β-galactosidase assay to study all nine published missense mutations. Figure 4A shows that the K103R, C238Y, K241E, and R271W mutations completely disrupt the binding with values fivefold less than WT, similar to that of the novel Q164X nonsense mutation (*P* < 0.01 for each comparison). The V49M change showed no effect (mean ± SD, 97 ± 1% of WT), whereas the R16H, I257V, A299V, and R304Q variants showed a small drop in binding compared to WT (39 ± 8%, 35 ± 25%, 58 ± 13%, and 76 ± 17% of WT, respectively).

We used a combination of clinical, experimental, and in silico methods to analyze how *AIP* mutations would change the AIP protein structure and function. Out of the known 49 *AIP* variants 35 result in a truncated or no protein (12 nonsense mutations, 12 frameshifts, six splice site, four large deletions, and one promoter mutation) [Tahir et al., 2010]. Previous studies convincingly showed that loss of the C-terminal end of the protein results in loss of function [Leontiou et al., 2008; Petrulis and Perdew, 2002]. The in-frame insertion mutation of exon 6 has been shown to disrupt the antiproliferative effect of AIP [Leontiou et al., 2008]. No functional data are available for the four in-frame deletions. The nine missense changes result in full-length proteins (R16H, V49M, K103R, C238Y, K241E, I257V, R271W, A299V, and R304Q). The location of these missense mutations are shown on a model of AIP, which was based on the structure of FKBP51 (Fig. 4B).

R16H has been identified in control subjects as well as sporadic and familial patients, and no LOH was shown in a tumor sample [Buchbinder et al., 2008; Cazabat et al., 2007; Daly et al., 2007; Georgitsi et al., 2007; Yaneva et al., 2008]. A reduction in PDE > A5 binding was observed, but this was less than threefold difference from WT. It is important to note that the PDE > A5-AIP interaction is at the C-terminal end of the molecule [Bolger et al., 2003]. Although the R16 amino acid is conserved in vertebrates, the R16H change probably represents a rare polymorphism as previously suggested [Raitila et al., 2007].

The V49M change was found in a sporadic childhood-onset somatotroph adenoma case, but no LOH was found in the tumor sample [Iwata et al., 2007]. The PDE4A5 binding data was similar to WT, as expected, as PDE4A5 binds the C-terminal of the AIP molecule while this variant affects the N-terminal of the protein. This change is conserved in most vertebrates, but not in lower species. Although no further data are available, the clinical significance of this variant is uncertain.

The K103R variant was identified in a patient with a childhood-onset corticotroph adenoma [Beckers et al., 2008]. Although the K > R is conservative substitution, the K103 residue is a conserved amino acid in most species and the PDE binding assay suggests a profoundly reduced activity (8 ± 5% of WT), supporting the possibility that this mutation has a functional impact.

The C238Y mutation was found in a Mexican family with three acromegalic brothers [Leontiou et al., 2008]. It disrupts a consensus TPR motif amino acid. To understand the relevance of this amino acid change we need to examine the structure of the TPR domains. AIP has three TPR motifs (Fig. 4B and C). Each TPR motif is composed of a pair of antiparallel helices, termed helices A and B. The consecutive helices of TPR domains are packaged together so each helix shares two immediate helix neighbors [Das et al., 1998]. A typical TPR consensus sequence pattern of small and large hydrophobic residues has been defined [D'Andrea and Regan, 2003; Sikorski et al., 1990]. Consensus amino acids (TPR residue positions marked as numbered diamonds on Fig. 4C) are located at position 4, 7, 8, and 11 in helix A and position 20, 24, 27, and 32 in helix B [D'Andrea and Regan, 2003; Sikorski et al., 1990]. Small hydrophobic residues are commonly observed at positions 8, 20, and 27 within the TPR motif. Residues 8 and 20 are located at the position of closest contact between the A and B helices of a TPR, whereas residue 27 on helix B is located at the interface of three helices (A, B, and the A helix of the next TPR motif) within a three-helix bundle [Das et al., 1998]. The C238Y mutation is at position 8 of the A helix of the second TPR domain of AIP and its mutation could destabilize the A and B helix packaging of the second TPR domain (Fig. 4C) [D'Andrea and Regan, 2003; Hidalgo-de-Quintana et al., 2008]. Both the cell proliferation [Leontiou et al., 2008] and the PDE binding assays (Fig. 4A) show that this mutant loses its activity in these two different types of functional assays. Therefore, all the data strongly suggest that this mutation has a functional impact.

The K241E mutation was identified in a FIPA family [Daly et al., 2007]. Interestingly, the same amino acid can also be affected by a stop mutation [Beckers et al., 2008]. This large conserved residue is part of the TPR sequence consensus at position 11 (Fig. 4C), and is suggested to be required for the packing of adjacent TPR α-helices [D'Andrea and Regan, 2003; Hidalgo-de-Quintana et al., 2008]. Our experimental data on protein interaction show a reduced ability to interact with PDE4A5 supporting the possible functional impact of the variant.

The I257V change affects a conserved small residue in most vertebrates in the B helix of the second TPR domain at position 27, which is thought to be important in the packaging of the adjacent helices [Das et al., 1998], in this case, A and B helices of TPR2 and A helix of TPR3 (Fig. 4C). It was found in a sporadic patient with unusual cell type (TSHoma) [Montanana et al., 2009]. A reduction in PDE4A5 binding was observed, but this was less than threefold difference from WT. LOH data are not available; therefore, the possibility that it has a functional impact cannot be excluded.

The R271W is the second most common mutational hotspot in the AIP gene. This mutation has been identified in three independent families [Daly et al., 2007; Jennings et al., 2009] as well as in a sporadic giant [Igreja et al., 2010]. It disrupts a conserved amino acid in the third TPR; it corresponds to an arginine in several other TPR proteins at the equivalent location such as in protein phosphatase 5 (PP5A), AIP-L1, FKBP51, and FKBP52 proteins (Fig. 4C). Interestingly, this amino acid was selected for mutation to alanine experimentally even before human mutations were identified in this gene [Bolger et al., 2003; Laenger et al., 2009]. Both the human mutation R > W (Fig. 4A) and the experimental mutation of R > A [Bolger et al., 2003] completely disrupts the AIP–PDE binding. These experimental and clinical data strongly support the pathogenic role of this mutation.

The A299V change was described in a sporadic acromegaly patient [Georgitsi et al., 2007] and in the current manuscript (see above). This variant, which is conserved in most vertebrates, is located at the beginning of the α-7 helix in the area that is relevant for PDE > A5 interaction but binding in vitro was less than threefold difference from WT. We identified this change in four subjects of a family where the R304X mutation was also detected. Two unaffected patients carried both changes; one unaffected patient carried only the A299V change while one young female carried only the A299V change and was diagnosed with a microprolactinoma at the age of 30 years. As AIP knockout mice are not viable [Lin et al., 2007], and the subjects with the compound heterozygote genotype with the certainly pathogenic R304X mutation are unaffected, this variant is unlikely to have a functional impact, although the patient with a possible phenocopy (small prolactinoma in a young female is not typical of AIP mutation patients) carrying this change makes the status of this variant uncertain.

The R304Q locus is part of a CpG island mutational hotspot. This amino acid change is relatively conservative changing a longer side chain positively charged amino acid to a slightly shorter, uncharged, hydrophilic residue in the α-7 helix. The amino acid is conserved in mammals, but not in lower species. Our protein interaction data show a less than threefold difference in PDE4A5 binding. The pathogenic role of R304Q, however, is overwhelmingly supported by the fact that it has been identified in several independent FIPA families as well as in sporadic patients [Cazabat et al., 2007; Georgitsi et al., 2007; Leontiou et al., 2008; Vargiolu et al., 2009] (and the current study).

In summary, our two-hybrid analysis of the missense mutations showed that they fell into two groups: mutations that were generally considered to have a functional impact had β-galactosidase activity values more than fivefold different from WT, whereas the mutants not generally considered to have a functional impact had β-galactosidase values less than threefold different from WT. Of note, none of the b-galactosidase values for the missense mutants fell between these two values, providing a clear distinction between functionally significant and functionally nonsignificant mutants in this PDE4A5-related assay.

## Discussion

We have analyzed clinical data and performed genetic and functional studies to investigate the possible functional impact of some of the reported *AIP* sequence changes. The main findings of this study are: (1) all our AIP-positive families have at least one member with GH/PRL-staining adenomas, have significantly higher number of subjects affected suggesting higher penetrance than in *AIP*-negative FIPA families, and have at least one childhood-onset case in the majority of the families. (2) Six novel *AIP* germline and five previously described mutations were identified using sequencing and MLPA, and our data support the suggestion that in addition to exon and exon–intron junction sequencing, study of the promoter area, and a technique suitable for the detection of large deletions, should be part of the DNA analysis of patients with FIPA. (3) Our data suggest a functional impact for the dibasic promoter mutation we have identified, as it causes decreased promoter activity compared to the WT promoter. In addition, we reveal that the cAMP–PKA signaling pathway positively regulates the activity of the *AIP* promoter. (4) The exonic synonymous mutations studied were shown to have a functional impact due to abnormal splicing or decreased *AIP* mRNA expression. (5) We combined clinical, in silico prediction and protein–protein binding assay data to characterize all the missense *AIP* changes identified in pituitary adenoma patients to help define those that may represent rare single nucleotide polymorphisms, and which may have a role in the disease process.

Previously described mutations affecting exon–intron junctions were hypothesized to cause abnormal AIP protein based on in silico predictions. Here we show actual data from patient cDNA samples regarding the consequences of the c.249G > T mutation, confirming the possible functional impact of this change. Studying another silent mutation (c.807C > T), we predicted the abnormal splicing of exon 6 due to the disrupted binding of the splicing regulatory proteins. As this abnormality affects the last exon, studying its role is not as straight forward as other splicing mutations at earlier exons. Although our search for alternative splice products using various sets of oligonucleotides did not reveal an alternative splice product, a minigene approach confirmed the lack of a normal exon 5–6 product and patient cDNA samples showed reduced AIP mRNA expression. We hypothesize that this mutation may either cause destabilization of the mRNA leading to reduced expression levels, or alternative splicing to a new isoform not detected by our RT-PCR assays.

*AIP* promoter regulation has not previously been studied. We suggest that the c. −270_−269 area is part of the basic promoter, as its disruption significantly reduces basal promoter activity in vitro. Based on prediction programs, the mutation at c.−270_−269 position would disrupt several transcription factor binding sites, including the zinc-finger protein *SP1*. *SP1* can be activated by the cAMP–PKA pathway [Rohlff et al., 1997], which is important in proliferation and hormone release of somatotroph cells: gain-of-function mutations in the α-subunit of the G-protein (*GNAS*) and loss-of-function mutations of the regulatory subunit of PKA (*PRKAR1A*) lead to deregulation of the cAMP–PKA pathway and play a role in somatotroph tumorigenesis. Here we show that the activity of the *AIP* promoter can be upregulated by the stimulation of this pathway and the effect is inhibited by a PKA inhibitor. This first appears to be a paradox, as sporadic somatotroph adenomas show an *overactive* PKA pathway [Bertherat et al., 1995]. However, our data are compatible with increased *AIP* mRNA and protein expression described previously [Jaffrain-Rea et al., 2009; Leontiou et al., 2008] and corresponds to the fact that low AIP levels do not play a role in sporadic somatotroph tumorigenesis when *AIP* mutations are not present [Leontiou et al., 2008].

PDEs can also regulate the PKA pathway, and PDE4A5 and PDE2A are known partners of AIP [Bolger et al., 2003; de Oliveira et al., 2007]. The AIP–PDE4A5 interaction was shown to be lost in the presence of *AIP* mutations [Leontiou et al., 2008]. Here we further studied all the described missense *AIP* changes as well as our novel nonsense mutation with a quantitative assay. We found that a stop mutation and four missense mutations (K103R, C238Y, K241E, and R271W) completely disrupted the PDE4A5 binding. Three of these affected amino acids are known to be important for TPR structure [Das et al., 1998], and two have been experimentally shown to disrupt the function of the AIP protein [Bolger et al., 2003; Laenger et al., 2009; Leontiou et al., 2008]. Clinical data suggest, although do not prove, that the R16H (found in normals as well), V49M (lack of LOH in tumor sample), and the A229V (also identified in unaffected subjects with compound heterozygosity for the pathogenic R304X mutation) changes might not have a functional impact, and this is supported by the weak or no effect on PDE4A5 binding. The I257V change affects a conserved amino acid at a location known to be important for the α-helix packaging of the TPR domains, and despite the weak effect on PDE4A5 binding a functional impact is possible. The pathogenic role of the R304Q variant is beyond doubt. The lack of profound reduction in PDE4A5 binding suggests that this part of the molecule may not be important in this PDE4A5–AIP interaction. On the other hand, missense variants can often act by altering the stability or folding the protein, rather than affecting amino acids directly involved in a protein–protein interaction. Clearly, the lack of profoundly reduced binding to PDE4A5 does not rule out other types of dysfunction of these variant AIP molecules, and further studies are needed to clarify whether the reduced or lack of AIP–PDE4A5 interaction as measured in this in vitro assay is important for the pituitary pathogenic process.

We identified two asymptomatic carrier subjects with two changes in the *AIP* gene—p.R304X and p.A299V—and showed that these variants are located on opposite parental chromosomes. It has previously been demonstrated that the p.R304X hotspot mutation disrupts the function of the AIP molecule. The p.A299V change (first described in a sporadic patient with acromegaly) [Georgitsi et al., 2007] involves an amino acid substitution of alanine to valine. This residue is not part of the TPR-domain consensus. The PDE4A5 binding data did not support a profound loss of function due to this missense mutation. Also, if this change has functional impact, our subjects (65 and 70 years, both asymptomatic) would be compound-heterozygotes for *AIP* mutations, whereas *AIP* knockout mice are not viable (they die at embryonic age e10.5–e14.5 due to cardiac malformations) [Lin et al., 2007], thus suggesting this change has no functional impact. We, however, identified a p.A299V female carrier with a microprolactinoma. Combining all these data with the fact that her phenotype is not typical of AIP mutation positive patients, her case may represent a phenocopy. The A299V change is a variant with unknow significance, and further studies are needed to confirm that it has no functional impact.

More than two-thirds of FIPA families do not have a detectable mutation in the *AIP* gene, and show differences in age of onset and level of penetrance as well as heterogeneity of tumor types compared to AIP positive patients, suggesting that there is a phenotypic difference between FIPA families with AIP mutations and FIPA families where the disease is caused by another gene or genes. Currently, work is being carried out to identify these new disease loci.

In summary, 31% of our FIPA families harbor *AIP* mutations. They show a predilection for GH/PRL-synthesizing adenomas, younger age of onset, and higher penetrance of disease compared to *AIP*-negative FIPA families. The *AIP* promoter is positively regulated by the cAMP–PKA pathway, and functional characterization of *AIP* mutations reveal that our promoter mutation decreases promoter activity, whereas some synonymous changes result in abnormal splicing or reduced AIP expression. Searching for large deletions using MLPA and analysis of clinical data and functional characterization of rare *AIP* changes helps to clarify the pathogenic role of AIP in patients with familial and sporadic pituitary adenomas, and assists in the appropriate genetic counseling and clinical management of these patients and their families.

## Appendix: List of International FIPA Consortium Members

Akker, Scott, London, UK; Atkinson, Brew, Belfast, Northern Ireland; Aylwin, Simon, London, UK; Baldeweg, Stephanie, London, UK; Bevan, John, Aberdeen, UK; Cheetham, Tim, Newcastle, UK; Chew, Shern, London, UK; Choudry, Kiran, Texas, USA; Clayton, Richard, Stoke-On-Trent, UK; Damjanovic, Svetozar, Belgrade, Serbia; Darzy, Ken, Milton Keynes, UK; Dattani, Mehul, London, UK; Davis, Julian, Manchester, UK; Drake, Will, London, UK; Dzeranova, Larisa, Moscow, Russia; Edén, Britt, Engström, Sweden; Eguchi, Kuniki, Hiroshima, Japan; Fica, Simona, Bucharest, Romania; Flanagan, Daniel, Plymouth, UK; Frohman, Lawrence, Chicago, Illinois, USA; Gadelha, Monica, Rio de Janeiro, Brazil; Gallego, Patricia, Brisbaine, Australia; Gláz, Edit, Budapest, Hungary; Goldman, James, Boston, Massachustts, USA; Goldstone, Tony, London, UK; Howlett, Trevor, Leicester, UK; Inder, Warrick, Melbourne, Australia; Iwata, Takeo, Tokushima, Japan; Kaplan, Felicity, Stevenage, UK; Karavitaki, Niki, Oxford, UK; Laws, Ed, Boston, Massachusetts, USA; Lechan, Ron, Boston, Massachusetts, USA; Levy, Miles, Leicester, UK; Matsuno, Akira, Chiba, Japan; Miljic, Dragana, Belgrade, Serbia; Modenesi, Silvia, Belo Horizonte, Brazil; Molitch, Mark, Chicago, Illinois, USA; Musat, Madâlina, Bucharest, Romania; Orme, Steve, Leeds, UK; Patócs, Attila, Budapest, Hungary; Popovic, Vera, Belgrade, Serbia; Powell, Michael, London, UK; Quinton, Richard, Newcastle, UK; Randeva, Harpal, Warwick, UK; Ribeiro de Oliveira JR, Antônio, Belo Horizonte, Brazil; Schöfl, Christof, Erlangen, Germany; Soares, Beatriz, Belo Horizonte, Brazil; Spada, Anna, Milan, Italy; Strasburger, Christian, Berlin, Germany; Swords, Francesca, Norwich, UK; Tsagarakis, Stylianos, Athens, Greece; Vaks, Vladimir, Oxford, UK; Wass, John A.H., Oxford, UK; Widell, Hakan, Gotheborg, Sweden; Yarman, Sema, Istanbul, Turkey; Yoshimoto, Katsuhiko, Tokushima, Japan.
