# Supplementary material for: Characterization of Aryl Hydrocarbon Receptor Interacting Protein (AIP) Mutations in Familial Isolated Pituitary Adenoma Families
Source: Hum Mutat. 2010 Aug;31(8):950–60. doi: 10.1002/humu.21292 (PMC3065644; doi:10.1002/humu.21292)
Supplement: Supplementary file 1 [file humu0031-0950-SD1.pdf]

Supp. Figure S1

Alignment of AIP and FKBP51

'*****' denotes identical amino acids, while '**:**' and '**.**' mark strongly or moderately similar amino acids.

```
AIP_human         --------------------MADIIARLREDGIQKRVIQEGRGELPDFQDGTKATFHYRT 40
FKBP51_human      MTTDEGAKNNEESPTATVAEQGEDITSKKDRGVLKIVKRVGNGEETPMIG-DKVYVHYKG 59
                                       .: *:  :: *: * * : *.** . : .  *. .**:

AIP_human         LHSDDEGTVLDDSRARGKPMELIIGKKFKLPVWETIVCTMREGEIAQFLCDIKHVVLYP- 99
FKBP51_human      KLSN--GKKFDSSHDRNEPFVFSLGKGQVIKAWDIGVATMKKGEICHLLCKPEYAYGSAG 117
                    *:  *. :*.*: *.:*: : :**   : .*:  *.**::***.::**. ::.   .

AIP_human         ------------------------------LVAKSLRNIAVGKDPLEGQ------RHCCG 123
FKBP51_human      SLPKIPSNATLFFEIELLDFKGEDLFEDGGIIRRTKRKGEGYSNPNEGATVEIHLEGRCG 177
                                                :: :: *:    .:* **       .  **

AIP_human         ------------VAQMREHSSLG--HADLDALQQNPQPLIFHMEMLKVESPG-------- 161
FKBP51_human      GRMFDCRDVAFTVGEGEDHDIPIGIDKALEKMQREEQCILYLGPRYGFGEAGKPKFGIEP 237
                              *.: .:*.     .  *: :*:: * :::      . ..*

AIP_human         -------------TYQQDPWAMTDEEKAKAVPLIHQEGNRLYREGHVKEAAAKYYDAIAC 208
FKBP51_human      NAELIYEVTLKSFEKAKESWEMDTKEKLEQAAIVKEKGTVYFKGGKYMQAVIQYGKIVSW 297
                                  ::.* *  :** : ..:::::*.  :: *:  :*. :* . ::

AIP_human         LKNLQMKEQPGSPEWIQLDQQITPLLLNYCQCKLVVEEYYEVLDHCSSILNKYDDNVKAY 268
FKBP51_human      LE---MEYGLSEKESKASESFLLAAFLNLAMCYLKLREYTKAVECCDKALGLDSANEKGL 354
                  *:   *:   .. *    :. : . :** . * * :.** :.:: *.. *.  . * *.

AIP_human         FKRGKAHAAVWNAQEAQADFAKVLELDPALAPVVSRELQALEARIRQKDEEDKARFRGIF 328
FKBP51_human      YRRGEAQLLMNEFESAKGDFEKVLEVNPQNK-AARLQISMCQKKAKEHNERDRRIYANMF 413
                  ::**:*:  : : :.*:.** ****::*    ..  ::.  : : :::*.*:   : .:*

AIP_human         SH------------------------------------------ 330
FKBP51_human      KKFAEQDAKEEANKAMGKKTSEGVTNEKGTDSQAMEEEKPEGHV 457
```

Supp. Table S1. Clinical and genetic data of 32 families with familial isolated pituitary adenoma

Family ID	Country of origin	Clinical diagnosis	Immunostaining of pituitary adenoma	Sex	Age at Diagnosis (yr)	Size of pituitary adenoma	Germline promoter, exon and exon-intron junction sequencing and GeneBank accession number	MLPA	Data for family members
1	UK	Prolactinoma	No surgery	Male	43	Macroadenoma	No mutation	Normal dosage	
		Prolactinoma	No surgery	Male	17	Microadenoma			
		Prolactinoma	No surgery	Female	23	Microadenoma			
2	UK	NFPA	Null cell	Male	60	Macroadenoma	No mutation	Normal dosage	
		NFPA	No surgery	Female	64	Macroadenoma			
3	Brazil	Cushing's disease	Pituitary adenoma	Female	21	Macroadenoma	No mutation	Not done	
		Prolactinoma	Pituitary adenoma	Female	34	Microadenoma			
4	Brazil	Prolactinoma	No surgery	Male	51	Microadenoma	No mutation	Normal dosage	
		Prolactinoma	No surgery	Male	47	Macroadenoma			
		Prolactinoma	No surgery	Female	35	Microadenoma			
**5**	**UK**	**Acromegaly**	**GH**	**Male**	**24**	**Macroadenoma**	**c.662dupC p.E222X 218178098**	**Normal dosage**	**One carrier free of disease age 80yr**
		**Acromegaly**	**GH**	**Male**	**28**	**Macroadenoma**			
6	Hungary	Prolactinoma	PRL	Male	51	Macroadenoma	No mutation	Normal dosage	
		Prolactinoma	No surgery	Male	46	Macroadenoma			
**7**	**Germany**	**Gigantism**	**GH**	**Female**	**23**	**Macroadenoma**	**c.490C>T p.Q164X 218178083**	**Normal dosage**	**One carrier free of disease age 56yr**
		**Gigantism**	**No surgery**	**Male**	**20**	**Macroadenoma**			
8	USA	Acromegaly	GH, TSH, FSH, LH, occ. PRL	Female	57	Macroadenoma	No mutation	Not done	
		NFPA	N/A	Male	47	Macroadenoma			
**9**[1]	**UK**	**NFPA**	**No surgery**	**female**	**17**	**Microadenoma**	No mutation	**c.1104-1109_279+578 Ex1_Ex2del**	Five carriers, free of disease at 68, 62, 36, 5 and 2yr. Two subjects at risk tested negative
		**Somato-mammotrophinoma**	**GH, PRL**	**female**	**29**	**2 Microadenomas**			
		**NFPA**	**occ. GH, PRL**	**Male**	**18**	**Macroadenoma**			

Supp. Table S1. Clinical and genetic data of 32 families with familial isolated pituitary adenoma

Family ID	Country of origin	Clinical diagnosis	Immunostaining of pituitary adenoma	Sex	Age at Diagnosis (yr)	Size of pituitary adenoma	Germline promoter, exon and exon-intron junction sequencing and GeneBank accession number	MLPA	Data for family members
		Gigantism	GH, PRL, LH, FSH Sparsely gran.	Male	18	Macroadenoma		EU872273	
10	Northern Ireland	Acromegaly	GH	Male	20	Macroadenoma	c.910C>T p.R304X AM236344	Normal dosage	Twelve carriers of R304X, free of disease at 70, 69, 66, 56, 40, 38, 38, 35, 29, 26, 24, 2yr, two of these subjects also carry A299V. One unaffected carrier of A299V. Nine subjects at risk tested negative.
		Gigantism	Necrotic tissue	Male	13	Macroadenoma			
		Prolactinoma ?phenocopy	No surgery	Female	30	Microadenoma	c.896C>T, p.A299V EF203235		
11	Sweden	Acromegaly	N/A	Female	31	Macroadenoma	No mutation	Normal dosage	
		Acromegaly	N/A	Male	N/A	N/A			
12	USA	Acromegaly	No surgery	Male	52	Microadenoma	No mutation	Normal dosage	
		NFPA	No surgery	Male	43	Microadenoma			
13	Serbia	NFPA	No surgery	Female	36	Microadenoma	No mutation	Not done	
		Prolactinoma	No surgery	Female	25	Microadenoma			
14	UK	Acromegaly	No surgery	Female	59	Microadenoma	No mutation	Normal dosage	
		Prolactinoma	No surgery	Female	28	Microadenoma			
		Prolactinoma	No surgery	Female	38	Microadenoma			
15	Japan	Acromegaly	GH	Male	59	Macroadenoma	No mutation	Normal dosage	
		Acromegaly	No surgery	Female	30	N/A			
16	Serbia	NFPA	LH,FSH, occ. TSH, PRL	Female	73	Macroadenoma	No mutation	Not done	
		NFPA	N/A	Male	40	Macroadenoma			
17[2]	Australia	Gigantism	Eosinophil adenoma, sparsely gran.	Male	13	Microadenoma	c.74_81delins7 p.L25PfsX130 218178084	Normal dosage	Three carriers, free of disease at 45, 21, 11yr
		Somato-mammotropinoma	Eosinophil adenoma	Male	38	Macroadenoma			

Supp. Table S1. Clinical and genetic data of 32 families with familial isolated pituitary adenoma

Family ID	Country of origin	Clinical diagnosis	Immunostaining of pituitary adenoma	Sex	Age at Diagnosis (yr)	Size of pituitary adenoma	Germline promoter, exon and exon-intron junction sequencing and GeneBank accession number	MLPA	Data for family members
		**Prolactinoma**	**PRL, occ. GH**	**Female**	**25**	**Macroadenoma**			
		**Acromegaly**	**Eosinophil adenoma**	**Male**	**27**	**Macroadenoma**			
		**Acromegaly**	**Basophil adenoma**	**Female**	**32**	**Macroadenoma**			
**18**	**USA**	**NFPA**	**occ. PRL TSH, LH, FSH**	**Female**	**52**	**Macroadenoma**	**c.911G>A p.R304Q EF203236**	**Normal dosage**	**no other sample available**
		**Prolactinoma**	**No surgery**	**Female**	**23**	**Macroadenoma**			
19	Brazil	NFPA	occ. FSH	Male	54	Macroadenoma	No mutation	Normal dosage	
		NFPA	occ. FSH,LH	Male	44	Macroadenoma			
20	Brazil	NFPA	ACTH (silent corticotroph adenoma)	Female	28	Macroadenoma	No mutation	Normal dosage	
		NFPA	N/A	Male	N/A	Macroadenoma			
21	Greece	Acromegaly	GH, occ. PRL	Female	42	Macroadenoma	No mutation	Normal dosage	
		Acromegaly	No surgery	Female	40	Macroadenoma			
22	UK	Acromegaly	GH, densely gran.	Female	36	Macroadenoma	No mutation	Normal dosage	
		Acromegaly	N/A	Female	N/A	N/A			
23	Serbia	Prolactinoma	No surgery	Male	40	Macroadenoma	No mutation	Normal dosage	
		Prolactinoma	No surgery	Female	N/A	Microadenoma			
24	Northern Ireland	Acromegaly	N/A	Male	44	Macroadenoma	No mutation	Normal dosage	
		Acromegaly	N/A	Male	32	Macroadenoma			
25	UK	Acromegaly	N/A	Female	50	Macroadenoma	No mutation	Normal dosage	
		Acromegaly	No surgery	Female	N/A	N/A			
		Gigantism	No surgery	Male	N/A	N/A			
26	Germany	Acromegaly	N/A	Male	N/A	Macroadenoma	No mutation	Normal dosage	
		Acromegaly	N/A	Male	N/A	N/A			
27	UK	Prolactinoma	No surgery	Female	26	Microadenoma	No mutation	Not done	

**Supp. Table S1.** Clinical and genetic data of 32 families with familial isolated pituitary adenoma									
**Family ID**	**Country of origin**	**Clinical diagnosis**	**Immunostaining of pituitary adenoma**	**Sex**	**Age at Diagnosis (yr)**	**Size of pituitary adenoma**	**Germline promoter, exon and exon-intron junction sequencing and GeneBank accession number**	**MLPA**	**Data for family members**
		Acromegaly	GH	Female	46	Macroadenoma			
**28**	**UK**	**Prolactinoma**	**Necrotic tissue**	**Male**	**14**	**Macroadenoma**	**c.249G>T p.G83AfsX15 218178082**	**Normal dosage**	**One carrier free of disease at the age of 75yr, six subjects at risk tested negative**
		**Prolactinoma**	**No surgery**	**Male**	**60**	**Macroadenoma**			
		**Gigantism**	**No surgery**	**Male**	**N/A**	**N/A**			
29	Turkey	Acromegaly	No surgery	Female	31	Macroadenoma	No mutation	Normal dosage	
		Gigantism	Eosinophil adenoma	Male	18	Macroadenoma			
**30**	**UK**	**Gigantism**	**GH**	**Female**	**6**	**Macroadenoma**	**c.910C>T p.R304X AM236344**	**Normal dosage**	**Two carriers free of disease at 73, 6yr. One subject at risk tested negative**
		**Acromegaly**	**N/A**	**Female**	**32**	**Macroadenoma**			
		**Acromegaly**	**N/A**	**Male**	**17**	**Macroadenoma**			
		**Gigantism**	**No surgery**	**Male**	**N/A**	**Macroadenoma**			
		**Prolactinoma**	**PRL**	**Female**	**16**	**Macroadenoma**			
31	UK	Acromegaly	GH	Female	17	Macroadenoma	No mutation	Normal dosage	
		Prolactinoma	No surgery	Female	38	Microadenoma			
32	UK	Acromegaly	N/A	Female	23	Macroadenoma	No mutation	Normal dosage	
		Prolactinoma	N/A	Female	25	Macroadenoma			
33	UK	Prolactinoma	No surgery	Female	25	Macroadenoma	No mutation	Normal dosage	
		Acromegaly	GH	Male	51	Macroadenoma			
34	UK	Prolactinoma	No surgery	Female	23	Microadenoma	No mutation	Normal dosage	
		Prolactinoma	No surgery	Female	30	Macroadenoma			
35	UK	NFPA	No surgery	Male	60	Macroadenoma	No mutation	Normal dosage	
		NFPA	N/A	Male	65	Macroadenoma			
36	Russia	Prolactinoma	No surgery	Female	44	Microadenoma	No mutation	Not done	
		Prolactinoma	No surgery	Female	12	Macroadenoma			
37	USA	Acromegaly	GH	Male	70	Macroadenoma	No mutation	Not done	
		Acromegaly	GH	Female	24	Macroadenoma			
**38**	**Northern**	**Gigantism**	**No surgery**	**Male**	**19**	**Macroadenoma**	**c.910C>T**	**Normal**	**3 carriers free of**

**Supp. Table S1.** Clinical and genetic data of 32 families with familial isolated pituitary adenoma									
**Family ID**	**Country of origin**	**Clinical diagnosis**	**Immunostaining of pituitary adenoma**	**Sex**	**Age at Diagnosis (yr)**	**Size of pituitary adenoma**	**Germline promoter, exon and exon-intron junction sequencing and GeneBank accession number**	**MLPA**	**Data for family members**
	**Ireland**	**Gigantism**	**GH**	**Female**	**13**	**Macroadenoma**	**p.R304X**<br>**AM236344**	**dosage**	**disease at age 68, 63 and 54y. Four subjects at risk tested negative**

[1]Family previously reported in (Georgitsi et al., 2007; Georgitsi et al., 2008)
[2]Family previously reported in (Pestell et al., 1989)
Nomenclature of cDNA and protein variants follows the Human Genome Variation Society guidelines (www.hgvs.org/mutnomen). Nucleotide numbering reflects cDNA numbering with +1 corresponding to the A of the ATG translation initiation codon in the reference sequence. The initiation codon is codon 1.
Families with AIP mutations are marked with bold letters.
MLPA, Multiplex-ligation dependent probe amplification; GH, growth hormone; PRL, prolactin; TSH, thyroid stimulating hormone; FSH, follicule-stimulating hormone; LH, luteinizing hormone; NFPA, non-functioning adenoma; N/A, not available; occ., occasionally; gran., granulated

Supp. Table S2 Updated clinical and genetic data of 26 families with familial isolated pituitary adenoma originally reported by Leontiou et al. (Leontiou et al., 2008)

Family ID	Country of origin	Clinical diagnosis	Immunostaining of pituitary adenoma	Sex	Age at diagnosis (yr)	Size of pituitary adenoma	Germline promoter, exon and exon-intron junction sequencing and GeneBank accession number	MLPA	Data for family members
**Family I[1]**	**Mexico**	**acromegaly**	**GH sparsely gran.**	**M**	**23**	**macroadenoma**	**c.713G>A p.C238Y EF643648**	**Not done**	**One carrier parent**
		**acromegaly**	**GH**	**M**	**21**	**macroadenoma**			
		**acromegaly**	**No surgery**	**M**	**19**	**macroadenoma**			
**Family II[1]**	**Brazil**	**gigantism**	**GH**	**M**	**17**	**macroadenoma**	**c.70G>T p.E24X EF643644**	**Normal dosage**	**Two carriers, one parent and one sibling not affected at the age of 28yr**
		**acromegaly**	**GH, PRL**	**M**	**24**	**macroadenoma**			
		**gigantism**	**GH**	**M**	**15**	**macroadenoma**			
		**acromegaly**	**GH**	**M**	**17**	**macroadenoma**			
		**gigantism**	**NA**	**M**	**13**	**macroadenoma**			
		**gigantism**	**NA**	**M**	**16**	**macroadenoma**			
		**gigantism**	**NA**	**M**	**18**	**macroadenoma**			
Family III[2]	USA	gigantism	No surgery	M	23	macroadenoma	No mutation	Normal dosage	
		acromegaly	Acidophil tu.	F	21	macroadenoma			
**Family IV[2]**	**Serbia**	**gigantism**	**GH sparsely gran.**	**M**	**20**	**macroadenoma**	No mutation	**Exon1-6 deletion**	**One carrier parent died not due to pituitary adenoma**
		**gigantism**	**GH sparsely gran.**	**F**	**10**	**macroadenoma**			
**Family V[2]**	**USA**	acromegaly	**GH, PRL sparsely gran.**	F	32	macroadenoma	**c.241C>T p.R81X EF643647**	**Normal dosage**	**Three carriers, free of disease at age 73,40,38yr**
		acromegaly	**GH, PRL sparsely gran.**	M	32	macroadenoma			

Supp. Table S2 Updated clinical and genetic data of 26 families with familial isolated pituitary adenoma originally reported by Leontiou et al. (Leontiou et al., 2008)

**Family VI[2,3]**	**Japan**	gigantism	**GH, PRL sparsely gran.**	F	14	macroadenoma	**c.[-270_-269CG>AA; -220G>A]** **EF643645**	**Normal dosage**	**One carrier parent free of disease**
		gigantism	**GH sparsely gran.**	F	10	macroadenoma			
Family VII[2]	Japan	acromegaly	GH	M	49	macroadenoma	No mutation	Not done	
		acromegaly	GH	M	24	macroadenoma			
Family VIII[2]	USA	acromegaly	GH sparsely gran.	M	39	macroadenoma	No mutation	Normal dosage	
			NA	M	38	macroadenoma			
Family IX[2]	Sweden	acromegaly	NA	F	71	macroadenoma	No mutation	Normal dosage	
			NA	F	70	macroadenoma			
			NA	M	48	macroadenoma			
			NA	M	14	microadenoma			
**Family X[2]**	UK	**gigantism acromegaly**	**GH sparsely gran.**	M	15	macroadenoma	**c.805_825dup** **p.F269_H275dup***	**Normal dosage**	**Four carriers not affected at age 55, 45, 31, 31yr**
		**gigantism**	**GH sparsely gran.**	M	29	macroadenoma			
		**acromegaly**	**GH sparsely gran.**	M	15	macroadenoma			
Family XI	UK	acromegaly prolactinoma	GH sparsely gran.	F	30	macroadenoma	No mutation	Normal dosage	
			No surgery	F	29	macroadenoma			
**Family XII**	**UK**	**acromegaly**	**GH sparsely gran.**	**F**	**42**	**macroadenoma**	**c.910C>T** **p.R304X** **AM236344**	**Normal dosage**	**no unaffected carriers (five potential carriers were screened and were negative)**
		**gigantism**	**GH sparsely gran.**	**F**	**24**	**macroadenoma**			
		**acromegaly**	**NA**	**F**	**24**	**macroadenoma**			
		**acromegaly**	**GH,PRL**	**F**	**27**	**macroadenoma**			
		**prolactinoma**	**PRL**	**M**	**25**	**macroadenoma**			
		**acromegaly**	**NA**	**M**	**NA**	**macroadenoma**			
		**acromegaly**	**NA**	**M**	**NA**	**macroadenoma**			
		**acromegaly**	**NA**	**M**	**NA**	**macroadenoma**			

Supp. Table S2 Updated clinical and genetic data of 26 families with familial isolated pituitary adenoma originally reported by Leontiou et al. (Leontiou et al., 2008)

Family XIII	UK	acromegaly	No surgery	F	68	macroadenoma	No mutation	Normal dosage	
			GH sparsely gran.	F	45	macroadenoma			
Family XIV	Brazil	acromegaly prolactinoma	GH sparsely gran.	M	41	macroadenoma	No mutation	Normal dosage	
			No surgery	F	25	macroadenoma			
Family XV	Finland	acromegaly	NA	F	54	macroadenoma	No mutation	Normal dosage	
				F	34	macroadenoma			
**Family XVI[4]**	**UK**	**acromegaly**	**no surgery**	F	47	macroadenoma	**c.807C>T p.= EF643649**	**Normal dosage**	**Two carriers not affected at the age of 51 and 29yr**
		**acromegaly**	**NA**	M	58	macroadenoma			
Family XVII[4]	UK	acromegaly	NA	F	52	macroadenoma	No mutation	Normal dosage	
			GH sparsely gran.	M	45	macroadenoma			
Family XVIII	UK	acromegaly	GH	M	43	macroadenoma	No mutation	Normal dosage	
			No surgery	M	46	macroadenoma			
Family XIX	Serbia	acromegaly	GH sparsely gran.	M	36	macroadenoma	No mutation	Normal dosage	
			NA	M	50	macroadenoma			
Family XX	UK	acromegaly	GH densely gran.	M	24	macroadenoma	No mutation	Normal dosage	
			NA	F	40	macroadenoma			
**Family XXI**	**UK**	**acromegaly**	**GH sparsely gran.**	**M**	**35**	**macroadenoma**	**No mutation**	**c.1-?_993+?del Exon1-6 deletion**	**Three carriers, 60, 57 and 80yr unaffected**
		**acromegaly**	**GH**	**M**	**19**	**macroadenoma**			
		**acromegaly**	**GH**	**M**	**23**	**macroadenoma**			
Family XXII	Malta	acromegaly	GH	F	53	macroadenoma	No mutation	Normal dosage	
			GH	F	54	macroadenoma			
			GH	M	58	macroadenoma			
Family XXIII	Serbia	acromegaly	GH	F	35	macroadenoma	No mutation	Normal	

Supp. Table S2 Updated clinical and genetic data of 26 families with familial isolated pituitary adenoma originally reported by Leontiou et al. (Leontiou et al., 2008)

			GH	F	36	macroadenoma		dosage	
**Family XXIV**	**Romania**	**acromegaly**	**GH, PRL**	F	17	macroadenoma	**c.910C>T p.R304X AM236344**	**Normal dosage**	**One carrier free of disease at 72yr**
		**prolactinoma/acromegaly**	**No surgery**	F	26	macroadenoma			
**Family XXV**	**Romania**	**acromegaly**	**GH, PRL, ACTH; FSH occ.**	F	30	macroadenoma	**c.911G>A p.R304Q EF203236**	**Normal dosage**	**No carrier identified as no other family member available for testing**
		**acromegaly**	**GH**	F	17	macroadenoma			
Family XXVI	UK	acromegaly	GH	M	30	macroadenoma	No mutation	Normal dosage	
		NFPA	No positive hormone staining	M	42	macroadenoma			

*This mutation has previously been annotated c.794_823dup, p.A274_H275ins10 (EF643650) (Leontiou et al., 2008) while the current appropriate annotation is c.805_825dup, p.F269_H275dup
[1]Family previously reported in (Gadelha et al., 1999; Gadelha et al., 2000)
[2]Family previously reported in (Soares et al., 2005)
[3]Family previously reported in (Matsuno et al., 1994)
[4]Family previously reported in (McCarthy et al., 1990)
Nomenclature of cDNA and protein variants follows the Human Genome Variation Society guidelines (www.hgvs.org/mutnomen). Nucleotide numbering reflects cDNA numbering with +1 corresponding to the A of the ATG translation initiation codon in the reference sequence. The initiation codon is codon 1.
Families with AIP mutations are marked with bold letters.
MLPA, Multiplex-ligation dependent probe amplification; GH, growth hormone; PRL, prolactin; TSH, thyroid stimulating hormone; FSH, follicule-stimulating hormone; LH, luteinizing hormone; ACTH, adrenocorticotropic hormone; NFPA, non-functioning adenoma; N/A, not available; occ., occasionally; gran., granulated
